# Determinants of Telemedicine Service Use Among Middle-Aged and Older Adults in Germany During the COVID-19 Pandemic: Cross-Sectional Survey Study

**DOI:** 10.2196/50938

**Published:** 2024-04-23

**Authors:** Ariana Neumann, Hans-Helmut König, André Hajek

**Affiliations:** 1 Department of Health Economics and Health Services Research, University Medical Center Hamburg-Eppendorf, Hamburg, Germany

**Keywords:** telemedicine, telehealth, digital health, service use, COVID-19

## Abstract

**Background:**

The occurrence of the COVID-19 pandemic demanded fast changes in the delivery of health care. As a result, significant growth in the use of telemedicine services occurred. Research, especially from nationally representative German samples, is needed to better understand determinants of telemedicine use.

**Objective:**

The purpose of this study was to identify determinants of telemedicine service use among middle-aged and older adults during the COVID-19 pandemic in Germany.

**Methods:**

Cross-sectional, nationally representative data were taken from the German sample of the Survey of Health, Ageing and Retirement in Europe (SHARE). The German Corona Survey 2 (n=2039), which was conducted between June and August 2021, was used for this study. Reporting experience with remote medical consultations during the COVID-19 pandemic served as the outcome measure. Associations with socioeconomic, psychological, social, health-related, and COVID-19–related determinants were examined using multiple Firth logistic regressions.

**Results:**

Psychological factors including feeling nervous, anxious, or on edge (odds ratio [OR] 1.61, 95% CI 1.04-2.50; *P*=.03), feeling sad or depressed (OR 1.62, 95% CI 1.05-2.51; *P*=.03) and feelings of loneliness (OR 1.66, 95% CI 1.07-2.58; *P*=.02) were positively associated with telemedicine use. Moreover, forgoing medical treatment because of being afraid of being infected by SARS-CoV-2 (OR 1.81, 95% CI 1.10-2.97; *P*=.02) and describing limitations because of a health problem as severe were positively associated with the outcome (OR 2.11, 95% CI 1.12-4.00; *P*=.02). Socioeconomic and social factors were not significantly associated with telemedicine use in our sample.

**Conclusions:**

Middle-aged and older individuals in Germany seem to use telemedicine services according to psychological needs and health limitations. Especially when psychological symptoms are experienced, telemedicine seems to be a promising service option in this age group. Future research is needed to confirm these initial findings in postpandemic circumstances.

## Introduction

Telemedicine has been a big part of the digital transformation of the health care sector. Multiple definitions of telemedicine have been introduced in the past [1]. The World Health Organization (WHO) Global Observatory for eHealth [2] identified the four key characteristics of telemedicine: (1) its purpose is to provide clinical support, (2) it is intended to overcome geographical barriers, connecting users who are not in the same physical location, (3) it involves the use of various types of information and communication technology, and (4) its goal is to improve health outcomes. Therefore, telemedicine includes synchronous (eg, videoconferencing, telephone) as well as asynchronous (eg, mobile apps) health services, which are delivered via electronic devices.

Telemedicine is a presumably promising method to provide health care in the future, as it can improve access to care, save costs, and close treatment gaps [3,4]. For example, it could be a potentially valuable tool when dealing with future shortages of physicians as well as the increased demand for health care services caused by population aging; underserved rural areas can also easily be reached through telemedicine [3,5]. In past research, telemedicine was found to be an effective and cost-effective service delivery model that can be equal to in-patient visits in a variety of specialties [3,6-9]. Additionally, practitioners (eg, physicians and psychotherapists), as well as patients with conditions covered by various specialties, were found to be greatly satisfied with this form of service [8-10]. Despite the clear advantages of telemedicine, it has not yet been widely implemented. Implementations have often been decelerated by limitations regarding reimbursement, as well as clinical, legal, cost, and social issues [11,12]. Telemedicine rates of use were gradually increasing over the previous years but remained at a low level [13].

The occurrence of the COVID-19 pandemic demanded fast changes in the delivery of health care. COVID-19 caused a major public health burden globally and it was essential to reduce in-person contacts to decrease further spreading of the virus [14]. Therefore, many nonessential appointments with physicians were canceled or postponed [15]. In a German population-based sample, this was the case for about one-third of respondents after containment measures were implemented in March 2020 [16]. Moreover, overall use of health care decreased in the first months of the pandemic, a phenomenon that was also observed in Germany [17].

Telemedicine appeared to be a key solution to major pandemic challenges. To facilitate the transformation to telemedicine, changes in infrastructure, reimbursement, and legal conditions were made worldwide. In Germany, legal efforts for the digital transformation of the health care system had already been made in November 2019 with the Digital Healthcare Act (Digitale-Versorgung-Gesetz) [18]. Consequently, it was easier for physicians to prescribe, deliver, and bill for telemedicine, and also for patients to use the services. In response to the pandemic, additional teleconsultation services were developed and regulations concerning video consultations were adapted [19,20]. Thus, telemedicine rates of use increased tremendously [21]. For example, the trend report of the Central Research Institute of Ambulatory Care in Germany reported that the number of video consultations increased from 743 conducted in December 2019 to 302,180 conducted in December 2020 [21]. Especially when there was limited access to in-person medical appointments during the pandemic in Germany, telemedicine services seemed to be a frequently used alternative, and satisfaction with the services was found to be moderately high [22,23]. Even though telephone services were used most frequently in Germany during the pandemic, a sharp increase was observed in the use of video consultations [24,25].

Besides technological, financial, organizational, and legal aspects, patient acceptance seems to be a crucial factor for the successful implementation of telemedicine services [26]. Patient characteristics that have been found to be associated with telemedicine use in past systematic reviews include, for example, age, gender, education, marital status, health status, and prior experience with computers and health technology [27,28]. A preliminary selective review of large-scale studies that were conducted during the pandemic in the United States found that telemedicine rates of use were higher among patients from urban areas, areas with greater broadband availability, and areas with higher prepandemic levels of telehealth use [29]. Moreover, being White; speaking English as first language; having health insurance, higher income, and greater disease burden; and being middle-aged were associated with greater use [29]. Nevertheless, more studies examining the use of telemedicine services and associated patient characteristics during pandemic times are needed.

Studies with samples from Germany, where telemedicine played a major role and was frequently used during the pandemic, are especially scarce. Few studies have looked at different German samples during the pandemic. While some of these studies examined large, nationally representative or quota-based samples [22,23,30], other studies only included convenience or smaller selective samples [31-33]. These studies identified potential socioeconomic (male sex, younger age, higher or lower education, living with a partner in the same household, having children younger than 18 years), psychosocial (increased loneliness, increased life satisfaction, severe psychological distress, frequent social isolation, lack of company), health-related (poor self-rated health), experience-related (higher electronic literacy, past use of telemedicine) and COVID-19–related (higher perceived severity of COVID-19 infection, having had COVID-19 infection, subjective COVID-19–related challenges, COVID-19–related cognitive preoccupation, anxiety, and worries) determinants that were positively associated with actual telemedicine use during the pandemic [22,23,30-33]. However, more studies including large nationally representative samples from Germany are needed to secure these initial findings. Moreover, the different categories of determinants, which were only partly included in single studies (eg, psychosocial or COVID-19–related determinants), should be explored further.

Middle-aged and older individuals represent the largest age group in the German population [34]. Considering population aging, the proportion of middle-aged and older adults will grow even further in the near future in Germany. Due to their increased need for health care (due to, eg, chronic conditions, frailty, and cognitive or functional decline) and potentially limited mobility, these age groups represent a major target group for future telemedicine services. Nevertheless, they seem to use telemedicine less often than other age groups [28]. Although past systematic reviews found that telemedicine is an effective and feasible service delivery model in older adults, it was also stated that further research was required to determine how services could be adapted to the individual needs of older patients [35-37]. Better understanding the telemedicine use behavior of middle-aged and older individuals could significantly contribute to increased use, as well as widespread acceptance and satisfaction with future telemedicine services. Therefore, this study aimed to explore determinants of patient use of telemedicine services in a nationally representative sample of middle-aged and older individuals during the COVID-19 pandemic in Germany.

## Methods

### Sample

Cross-sectional data for this study were taken from the Corona Survey 2 [38] of the Survey of Health, Ageing and Retirement in Europe (SHARE) study [39]. SHARE is a multidisciplinary and cross-national panel study that explores health, social, economic, and environmental policies in individuals aged 50 years or older and their partners (regardless of age) from 26 European countries, Switzerland, and Israel. Starting in 2004, SHARE has so far conducted 8 waves. In each wave, new respondents are added to the sample to compensate for attrition. In response to the global COVID-19 pandemic, a special Corona Survey, to examine the health-related and socioeconomic impact of COVID-19, was introduced in June 2020. In the course of this survey, the usual computer-assisted personal interviews were replaced by telephone-administered interviews.

Participation rates for waves 1 to 8 and the Corona Survey 1 have been provided by SHARE [40,41]. According to Bergmann et al [40], these rates increased over time and reflect high overall panel stability. The final rates for the Corona Survey 2 are not available yet; however, an average retention rate of 86% (excluding recovery of respondents) was confirmed by SHARE user support. Due to the fact that SHARE assessed telemedicine service use for the first time in the Corona Survey 2, only data from this survey, which was conducted from June until August 2021, were included in our analyses. Moreover, only the German subsample (n=2039) was considered, which was done to promote comparability among participants due to existing heterogeneity regarding characteristics of health care systems, telemedicine regulations, and telemedicine use across the different countries [20,42,43]. For example, whereas countries such as Denmark, Italy, and Germany are described as advanced in telemedicine use trends, countries like Poland, Portugal, or Slovakia are still developing in the telemedicine field since the pandemic [42].

### Ethical Considerations

Verbal informed consent was collected from all individuals that participated in the telephone-administered interviews for the Corona Survey 2. The SHARE project has been repeatedly reviewed and approved by the ethics committee of the University of Mannheim (waves 1-3) and the Ethics Council of the Max Planck Society (waves 4-9; most recently in June 2021 with the ethics approval number 2021_24).

### Dependent Variable

In the SHARE Corona Survey 2, telemedicine service use by middle-aged and older adults during the pandemic was assessed with a metric variable: “Since the outbreak of Corona, how many remote medical consultations over the phone, computer, or any other electronic means, did you have, if any, with or without video?” Therefore, this study did not focus on one specific form of telemedicine or patient group and included consultations on online platforms (eg, video calls), as well as telephone appointments. The response format in the original questionnaire was numerical (ie, the number of experienced telemedicine consultations). For the sake of this analysis, this item was dichotomized (1=one or more remote medical consultations since the outbreak of the COVID-19 pandemic; 0=no use) because of small case numbers.

### Independent Variables

Independent variables were chosen in line with former research and based on theoretical considerations. Previous systematic reviews identified mostly socioeconomic (eg, sex, age, education, relationship status, area lived in) and health (eg, disease burden, psychological symptoms) determinants of telemedicine use [27-29]. Moreover, we considered the pandemic context, including the pandemic and social consequences, when choosing independent variables. Therefore, socioeconomic, psychological, social, health-related, and COVID-19–related factors were taken into account to explore their potential relationships with telemedicine service use. Socioeconomic factors that were included were sex, age, area lived in (big city, suburbs or outskirts of a big city, large town, small town, rural area or village), living with a partner in the same household (yes or no), employment status (retired, employed or self-employed, unemployed, or other) and the household’s ability to make ends meet regarding their total monthly income (with great difficulty or some difficulty, fairly easily, easily). Included psychological factors were feeling nervous, anxious, or on edge in the last month (yes or no), feeling sad or depressed in the last month (yes or no), and feeling lonely in the last month (yes or no). Furthermore, social factors included social and electronic contact frequency with people other than relatives (never, less than once a week, about once a week, several times a week, daily). Concerning health-related factors, having trouble sleeping recently (yes or no), the number of physical illnesses (including hip fracture; diabetes or high blood sugar; high blood pressure or hypertension; a heart attack, including myocardial infarction or coronary thrombosis; any other heart problem, including congestive heart failure; chronic lung diseases, such as chronic bronchitis or emphysema; and cancer or malignant tumor, including leukemia or lymphoma, but excluding minor skin cancers), limitations because of a health problem in usual activities (not limited; limited, but not severely; severely limited) and self-rated health (excellent, very good, good, fair, poor) were inspected. COVID-19–related factors that were included in the analyses were having received the COVID-19 vaccination (yes or no), oneself or anyone close having tested positive for COVID-19 (yes or no), forgoing medical treatment because of being afraid to become infected by SARS-CoV-2 (yes or no), and taking drugs or medicine as prevention against COVID-19 (yes or no).

### Statistical Analysis

First, sample characteristics were computed. Second, Firth logistic regressions were conducted to identify determinants of telemedicine service use during the pandemic. The Firth method was used to reduce small-sample bias, considering the small case numbers for some of the variables [44]. Due to high correlations, the variables regarding psychological symptoms (including feeling nervous, anxious, or on edge; feeling sad or depressed; and feeling lonely in the last month) were entered separately into the model. For sensitivity analyses, conventional multiple logistic regressions were also performed. Moreover, we computed additional analyses with age as a categorical variable (40-64 years, 65-74 years, ≥75 years) to test for a nonlinear relationship with the outcome. Odds ratios (ORs) are presented with the 95% CI. *P* values were considered statistically significant at an α level of <.05. Since the number of missing values for the independent variables was very small (below 1%), we did not use imputation techniques. Small levels of missing values are usually less likely to significantly bias results [45,46]. Therefore, listwise deletion was applied. Stata (version 16.1; StataCorp) was used for all statistical analyses.

## Results

### Sample Characteristics

The total sample consisted of 2039 individuals. The sample characteristics for all included variables are presented in Table 1. Overall, 54.2% (1105/2039) of the sample were women. The mean age of the participants was 70.6 (SD 8.7) years. Considering telemedicine service use during the pandemic, 5.7% (115/2031) of the sample reported that they had had remote medical consultations at least once.

**Table 1.** Sample characteristics (N=2039).

Characteristics		Values
**Telemedicine service use, n (%)**		
	Never	1916 (94.3)
	At least once	115 (5.7)
**Sex, n (%)**		
	Male	934 (45.8)
	Female	1105 (54.2)
Age (years), mean (SD)		70.6 (8.7)
**Age (years), n (%)**		
	40-64	573 (28.1)
	65-74	1083 (53.1)
	≥75	383 (18.78)
**Area lived in, n (%)**		
	Big city	289 (14.3)
	Suburbs or outskirts of a big city	177 (8.8)
	Large town	198 (9.8)
	Small town	549 (27.2)
	Rural area or village	807 (40)
**Living with partner in the same household, n (%)**		
	Yes	1521 (74.6)
	No	518 (25.4)
**Employment situation, n (%)**		
	Retired	1445 (70.9)
	Employed or self-employed	457 (22.4)
	Other	135 (6.6)
**Households’ ability to make ends meet, n (%)**		
	With great or some difficulty	151 (7.4)
	Fairly easily	599 (29.6)
	Easily	1277 (63)
**Nervous, anxious, or on edge in the last month, n (%)**		
	Yes	524 (25.8)
	No	1507 (74.2)
**Sad or depressed in the last month, n (%)**		
	Yes	609 (30)
	No	1419 (70)
**Feelings of loneliness in the last month, n (%)**		
	Yes	442 (21.8)
	No	1583 (78.2)
**Frequency of social contacts with nonrelatives, n (%)**		
	Never	229 (11.3)
	Less than once a week	583 (28.7)
	About once a week	452 (22.3)
	Several times a week	452 (22.3)
	Daily	312 (15.4)
**Frequency of electronic contacts with nonrelatives, n (%)**		
	Never	253 (12.5)
	Less than once a week	617 (30.4)
	About once a week	505 (24.9)
	Several times a week	504 (24.8)
	Daily	150 (7.4)
**Having trouble sleeping recently, n (%)**		
	Yes	686 (33.8)
	No	1344 (66.2)
Number of physical illnesses (range 0-6), mean (SD)		1.1 (1)
**Health limitations, n (%)**		
	Severely limited	310 (15.2)
	Limited, but not severely	744 (36.6)
	Not limited	981 (48.2)
**Self-rated health, n (%)**		
	Excellent	82 (4)
	Very good	320 (15.7)
	Good	839 (41.2)
	Fair	629 (30.9)
	Poor	166 (8.2)
**Received COVID-19 vaccine, n (%)**		
	Yes	189 (9.3)
	No	1847 (90.7)
**Self or anyone close tested positive for COVID-19, n (%)**		
	Yes	592 (29.1)
	No	1441 (70.9)
**Forgone medical treatment since COVID-19 pandemic, n (%)**		
	Yes	225 (11.1)
	No	1810 (88.9)
**Took drugs/medicine as prevention against COVID-19, n (%)**		
	Yes	45 (2.2)
	No	1990 (97.8)

### Regression Analysis

**Figure 1.**
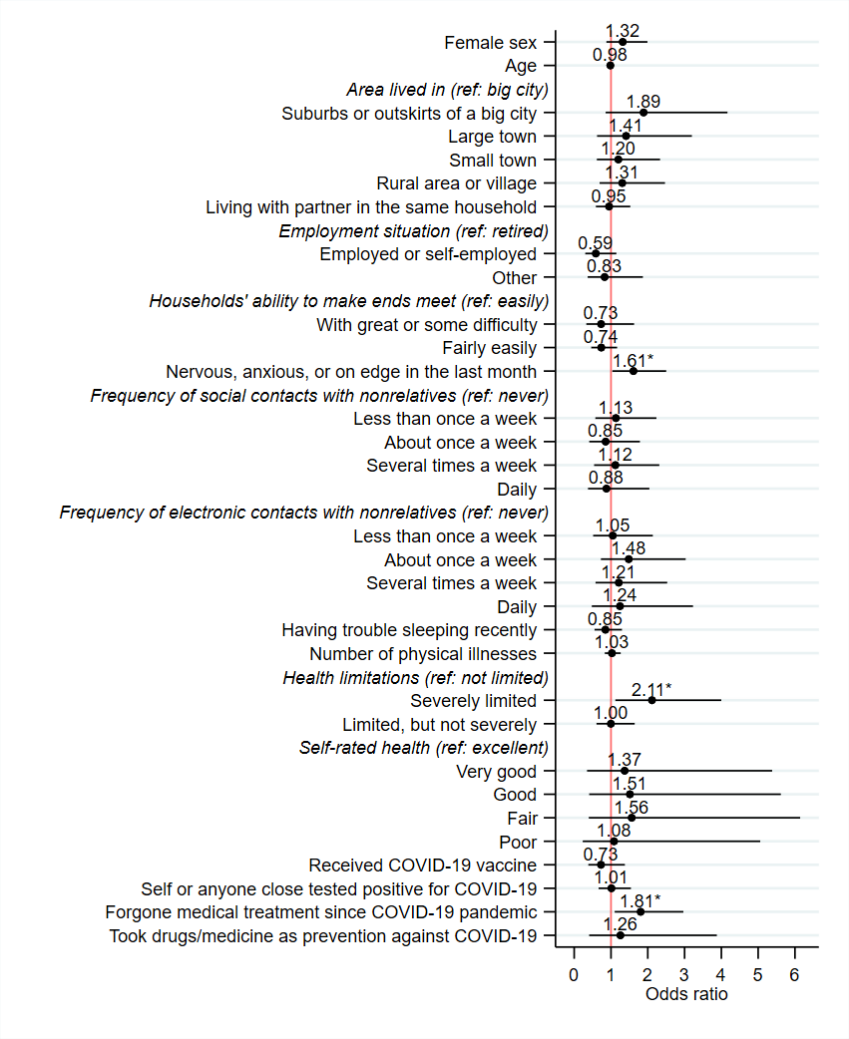
Results from Firth logistic regression for determinants of telemedicine service use during the COVID-19 pandemic. The model includes anxiety symptoms. Numbers represent odds ratios, with the 95% CIs shown as bars. More detailed results are provided in Table S1 in Multimedia Appendix 1. ref: reference category. *P<.05.

The analytic sample for the Firth logistic regressions included 1976 individuals. Results for the model, including anxiety symptoms, are presented in Figure 1 (see Multimedia Appendix 1 for detailed results for all models). Most of the included independent variables were not significantly associated with the outcome (*P*>.05). However, some associations were observed. Psychological factors, including feeling nervous, anxious, or on edge (OR 1.61, 95% CI 1.04-2.50; *P*=.03), feeling sad or depressed (OR 1.62, 95% CI 1.05-2.51; *P*=.03) and feeling lonely (OR 1.66, 95% CI 1.07-2.58; *P*=.02) in the last month were positively associated with the likelihood of telemedicine service use during the pandemic. Moreover, forgoing medical treatment because of being afraid to become infected by SARS-CoV-2 was positively associated with the outcome (OR 1.81, 95% CI 1.10-2.97; *P*=.02). Describing limitations because of health problems in the last 6 months as severe was also positively associated with the likelihood of telemedicine use during the pandemic (OR 2.11, 95% CI 1.12-4.00; *P*=.02).

In a sensitivity analysis in which Firth logistic regressions were replaced by conventional logistic regressions, similar associations were observed (see Tables S1-S3 in Multimedia Appendix 1 for detailed results). When age was included as a categorical variable in the models (see Table S4 in Multimedia Appendix 1 for detailed results), the youngest age group (40-64 years) was significantly more likely to use telemedicine services compared to the older age groups in our sample (65-74 and ≥75 years). Moreover, in these models, being employed or self-employed versus retired achieved statistical significance and was negatively associated with telemedicine use.

## Discussion

### Key Findings

This study aimed to identify determinants of telemedicine service use in a nationally representative sample of middle-aged and older adults during the COVID-19 pandemic in Germany. Based on data from the German sample of the SHARE Corona Survey 2, some associations of patient characteristics with telemedicine use were identified. This partly included health, psychological, and COVID-19–related factors. Socioeconomic and social determinants were not significantly associated with the outcome in this sample. So far, there has been limited research on determinants of telemedicine use in German samples. Our study findings thus extend our current knowledge regarding socioeconomic, social, health, psychological, and COVID-19–related determinants.

### Relation to Previous Research

Whereas none of the included socioeconomic determinants were associated with telemedicine service use in our sample, some recent studies identified significant relationships. Findings regarding associations of sex and age with telemedicine use were mixed in recent studies conducted during the pandemic in Germany, with some studies observing higher rates of use in male and younger individuals [22,23,30-32]. When observing age groups in our sample, older age groups (65-74 and ≥75 years) were less likely to use telemedicine. While our study did not indicate a significant association, Hajek and König [30] observed that middle-aged and older individuals who reported living with a partner in the same household were more likely to have participated in online consultations with physicians or therapists during the pandemic. These mixed findings could potentially be explained by variations in outcomes (eg, web-based consultations vs mobile app use), samples (eg, all age groups vs only those middle-aged and older), and time frames (2020 vs 2021 vs 2022) of the different studies. This clearly highlights the need for further studies on sociodemographic determinants in German samples. Similar to our results, employment status, financial situation, and area lived in were not significantly associated with telemedicine use in other German samples during the pandemic [23,30]. However, this is in contrast to research from the United States regarding telemedicine use during the pandemic [29,47]. This contrast may be explained by a larger variation in state-specific telehealth policies before and during the pandemic [48], as well as access factors, such as possession of digital devices or availability of high speed internet [49] in the United States compared to the German samples. In contrast to Germany, health care insurance is not obligatory in the United States and additional costs arise for uninsured individuals [50], which could have contributed to telemedicine use disparities caused by socioeconomic factors in the United States [49,51-53]. Further attention should be given to the impact of socioeconomic factors on telemedicine use in future research, especially with respect to postpandemic changes and the increasing availability of in-person visits.

Our study is one of very few that has examined the association of social determinants (ie, electronic and social contact frequency) and telemedicine service use. These determinants were not significantly associated with telemedicine use in our sample. This could mean that middle-aged and older adults used telemedicine services during the pandemic based more on health factors than on reduced social contact. Nevertheless, Rauschenberg et al [33] observed that telemedicine use was higher among young individuals who reported higher perceived social isolation and lack of company during the pandemic in Germany. These contrasting findings may imply that younger individuals have used telemedicine more frequently to deal with reduced social contact during the pandemic.

Furthermore, we found that perceiving one’s limitations because of a health problem as severe was associated with telemedicine service use. This suggests that individuals with severe health limitations preferentially used telemedicine services during the pandemic. Likewise, Hajek and König [30] found a significant association of poor self-rated health and telemedicine use during the pandemic in Germany. Additionally, a positive association of disease burden and telemedicine use was observed by Harju and Neufeld [29] in large-scale US samples during the pandemic. Potential reasons for that could include the (urgent) need for treatment, limited mobility, or precautions due to high risk of severe illness from COVID-19. Patients might have used telemedicine because of health needs and lack of in-person services during the pandemic. In contrast, we found that the number of physical illnesses and self-rated health were not associated with telemedicine use during the pandemic in our sample. A potential reason for that could be that these determinants may not reflect the actual need for medicine or telemedicine services. For example, having ever received a diagnosis of physical illnesses such as hip fracture, high blood sugar, or high blood pressure does not indicate that there currently is a higher need for treatment. Other studies that observed German samples during the pandemic also found a nonsignificant association of the presence of chronic conditions and telemedicine use [22,23]. Moreover, necessary treatment for patients with severe diseases (eg, physical examination, cancer treatment) was potentially more likely to be in person and still available during the pandemic. Future telemedicine services might be less suitable for these patient groups. Further research is needed to gain a better understanding of the possible impact of physical illness on telemedicine service use, especially in German samples.

Since few recent studies have examined the association of psychological symptoms with telemedicine use, our findings contribute to existing knowledge concerning psychological determinants during the pandemic in Germany. We observed that symptoms of anxiety, depression, or loneliness increased the likelihood of telemedicine use in middle-aged and older adults. Similar to our results, Hajek and König [30] observed a significant positive association of loneliness and telemedicine use in middle-aged and older adults during the pandemic in Germany. Likewise, Rauschenberg et al [33] found that psychological distress was associated with the current use of mobile health apps in a representative sample of youth aged 16-25 years from the German general population. Other studies with samples from the United States also observed a positive relationship of psychological symptoms with telemedicine use during pandemic times [54,55]. Therefore, it may be the case that findings regarding higher health care use in individuals with mental illness [56-60] can be applied to the field of telemedicine and the pandemic context. These initial findings illustrate the future potential of telemedicine in the field of mental health for middle-aged and older patients, since those who experienced psychological symptoms appeared to preferentially opt for telemedicine services. Moreover, mental health problems, such as anxiety or depression, have been shown to be positively associated with fear of COVID-19 [61]. This fear could also favor increased telemedicine use due to concerns of being infected with SARS-CoV-2 during in-person health care visits—this association was also found in our sample.

Additionally, other COVID-19–related determinants that were included in our sample (ie, vaccine status, COVID-19 infection history in oneself or close others, and preventive medication), were not significantly associated with telemedicine use. This is in contrast with findings from German samples that looked at younger and adult individuals during the pandemic [23,32,33] and found significant associations of COVID-19–related factors with telemedicine use (ie, higher perceived severity of COVID-19 infection; having had COVID-19 infection; subjective COVID-19–related challenges; and COVID-19–related cognitive preoccupation, anxiety, and worries). However, when looking at a similar sample to our study, which consisted of middle-aged and older adults during the pandemic in Germany, Hajek and König [30] did not find significant associations of COVID-19–related factors with telemedicine service use. This could potentially mean that middle-aged and older individuals’ decision to use telemedicine was less influenced by COVID-19–related factors than in the general adult or younger German population.

### Strengths and Limitations

This study is one of only a few studies that explore determinants of use of remote medical consultations in German middle-aged and older adults. The nationally representative sample of the widely acknowledged SHARE panel study provides insight into the largest age group of the German population. The data were collected during the COVID-19 pandemic and therefore account for the unique circumstances that individuals were faced with during that time.

However, some limitations should be considered. Telemedicine service use was measured using only one item, which indicated experience with remote medical consultations over the phone, computer, or any other electronic means since the outbreak of the pandemic. Therefore, we did not differentiate between different patient groups, telemedicine modalities, or frequency of use. This should be explored further in future studies. Furthermore, the survey covered a limited selection of socioeconomic, health, and psychosocial aspects. Future studies should include more extensive instruments and variables to make more reliable and comprehensive conclusions. In addition, the majority of our sample did not use telemedicine and case numbers were small for some of the included determinants. This lack of statistical power might explain why some of the tested relationships did not reach statistical significance. Consequently, future studies with very large German samples are needed. Furthermore, this analysis was based on self-reported cross-sectional data, and it is therefore difficult to identify causal relationships. Finally, we only focused on the German context. Future research should also consider cross-cultural differences in use and determinants of telemedicine to better understand potential barriers and facilitators in different cultural contexts and improve worldwide implementations.

### Conclusions

To achieve high rates of use and widespread acceptance of telemedicine, it is essential to understand determinants of telemedicine service use in middle-aged and older individuals. Our study findings stress the link between psychological symptoms and telemedicine use in Germany during the COVID-19 pandemic. Middle-aged and older adults seem to have used telemedicine services according to psychological needs and health limitations. One may conclude that, especially when they had psychological symptoms, middle-aged and older individuals accepted telemedicine as a service option. While socioeconomic and social factors were not associated with telemedicine service use, the associations of other health- and COVID-19-related determinants with use behavior remain unclear.

Future (longitudinal) studies are therefore required to confirm these initial findings and clarify whether they also apply to postpandemic circumstances, where widespread in-person visit availability returned. Some patients might have used telemedicine only because they had no other option. However, remote consultations might be especially suited for specific patient groups or forms of treatment and will remain part of postpandemic routine care. Furthermore, use of (remote) blended therapy might increase in the postpandemic context, as it combines the strengths of remote and in-person visits and can be adapted to individual patient preferences. Moreover, potential differences in determinants of telemedicine use between different service types (eg, asynchronous vs synchronous services) or patient groups (eg, mental health vs oncology patients) should be further investigated. Finally, it remains to be explored to what extent determinants of telemedicine use differ from determinants of general health care use, which could help to identify target groups and appropriate fields of application for future telemedicine services. This could be examined in the postpandemic context where both forms of services, in-person and telemedicine visits, are likely to be available to patients.

## Supplementary material

10.2196/50938 Multimedia Appendix 1 Results from logistic regression and Firth logistic regression for determinants of telemedicine service use during the COVID-19 pandemic.

## Abbreviations

OR: odds ratio
SHARE: Survey of Health, Ageing and Retirement in Europe
WHO: World Health Organization
