# Supplementary material for: Determinants of Telemedicine Service Use Among Middle-Aged and Older Adults in Germany During the COVID-19 Pandemic: Cross-Sectional Survey Study
Source: JMIR Aging. 2024 Apr 23;7:e50938. doi: 10.2196/50938 (PMC11063582; doi:10.2196/50938)
Supplement: Multimedia Appendix 1 [file aging-v7-e50938-s001.docx]

**Multimedia Appendix 1**

**Table S1. Results from logistic regression and Firth logistic regression for determinants of telemedicine service use during the COVID-19 pandemic (with anxiety symptoms).**

|  | Results from | Results from |
| --- | --- | --- |
| Independent Variables | Logistic Regression | Firth Logistic Regression |
|  |  |  |
| Female sex (ref: male) | 1.33 | 1.32 |
|  | (0.88 - 2.02) | (0.87 - 1.99) |
| Age | 0.98 | 0.98 |
|  | (0.95 - 1.01) | (0.95 - 1.01) |
| Area lived in (ref: big city) |  |  |
| Suburbs or outskirts of a big city | 1.92 | 1.89 |
|  | (0.85 - 4.34) | (0.86 - 4.17) |
| Large town | 1.41 | 1.41 |
|  | (0.61 - 3.29) | (0.62 - 3.20) |
| Small town | 1.23 | 1.20 |
|  | (0.62 - 2.42) | (0.62 - 2.33) |
| Rural area or village | 1.34 | 1.31 |
|  | (0.70 - 2.58) | (0.69 - 2.47) |
| Living with partner in the same household (ref: no) | 0.96 | 0.95 |
|  | (0.59 - 1.55) | (0.59 - 1.52) |
| Employment situation (ref: retired) |  |  |
| Employed or self-employed | 0.57 | 0.59 |
|  | (0.29 - 1.12) | (0.30 - 1.15) |
| Other | 0.79 | 0.83 |
|  | (0.34 - 1.83) | (0.37 - 1.87) |
| Households’ ability to make ends meet (ref: easily) |  |  |
| With great or some difficulty | 0.69 | 0.73 |
|  | (0.30 - 1.58) | (0.33 - 1.63) |
| Fairly easily | 0.72 | 0.74 |
|  | (0.45 - 1.16) | (0.46 - 1.17) |
| Nervous, anxious, or on edge in the last month (ref: no) | 1.63* | 1.61* |
|  | (1.04 - 2.55) | (1.04 - 2.50) |
| Frequency of social contacts with nonrelatives (ref: never) |  |  |
| Less than once a week | 1.17 | 1.13 |
|  | (0.58 - 2.34) | (0.57 - 2.23) |
| About once a week | 0.87 | 0.85 |
|  | (0.41 - 1.84) | (0.41 - 1.79) |
| Several times a week | 1.14 | 1.12 |
|  | (0.54 - 2.41) | (0.54 - 2.31) |
| Daily | 0.88 | 0.88 |
|  | (0.37 - 2.09) | (0.38 - 2.04) |
| Frequency of electronic contacts with nonrelatives (ref: never) |  |  |
| Less than once a week | 1.08 | 1.05 |
|  | (0.52 - 2.24) | (0.51 - 2.14) |
| About once a week | 1.54 | 1.48 |
|  | (0.74 - 3.20) | (0.73 - 3.03) |
| Several times a week | 1.25 | 1.21 |
|  | (0.59 - 2.65) | (0.58 - 2.53) |
| Daily | 1.24 | 1.24 |
|  | (0.46 - 3.32) | (0.48 - 3.23) |
| Having trouble sleeping recently (ref: no) | 0.84 | 0.85 |
|  | (0.54 - 1.30) | (0.55 - 1.30) |
| Number of physical illnesses (range 0-6) | 1.02 | 1.03 |
|  | (0.83 - 1.27) | (0.83 - 1.27) |
| Health limitations (ref: not limited) |  |  |
| Severely limited | 2.14* | 2.11* |
|  | (1.12 - 4.09) | (1.12 - 4.00) |
| Limited, but not severely | 0.99 | 1.00 |
|  | (0.60 - 1.65) | (0.61 - 1.64) |
| Self-rated health (ref: excellent) |  |  |
| Very good | 1.64 | 1.37 |
|  | (0.36 - 7.45) | (0.35 - 5.38) |
| Good | 1.85 | 1.51 |
|  | (0.43 - 7.95) | (0.41 - 5.62) |
| Fair | 1.91 | 1.56 |
|  | (0.42 - 8.65) | (0.40 - 6.14) |
| Poor | 1.29 | 1.08 |
|  | (0.24 - 6.88) | (0.23 - 5.06) |
| Received COVID-19 vaccine (ref: no) | 0.74 | 0.73 |
|  | (0.38 - 1.43) | (0.38 - 1.38) |
| Self or anyone close tested positive for COVID-19 (ref: no) | 1.00 | 1.01 |
|  | (0.65 - 1.54) | (0.66 - 1.54) |
| Forgone medical treatment since COVID-19 pandemic (ref: no) | 1.81* | 1.81* |
|  | (1.09 - 3.01) | (1.10 - 2.97) |
| Took drugs/medicine as prevention against COVID-19 (ref: no) | 1.11 | 1.26 |
|  | (0.33 - 3.75) | (0.41 - 3.88) |
| Constant | 0.08+ | 0.11 |
|  | (0.00 - 1.59) | (0.01 - 2.01) |
|  |  |  |
| Observations | 1,976 | 1,976 |
| Pseudo R² | 0.045 | 0.052 |
| Likelihood ratio chi-square test statistic | 38.69 |  |
| Wald chi-square test statistic |  | 39.31 |

*Note.* Odds Ratios are reported with 95% confidence intervals in parentheses. * p<0.05, + p<0.10.

**Table S2. Results from logistic regression and Firth logistic regression for determinants of telemedicine service use during the COVID-19 pandemic (with depressive symptoms).**

|  | Results from | Results from |
| --- | --- | --- |
| Independent Variables | Logistic Regression | Firth Logistic Regression |
|  |  |  |
| Female sex (ref: male) | 1.33 | 1.32 |
|  | (0.88 - 2.03) | (0.88 - 1.99) |
| Age | 0.98 | 0.98 |
|  | (0.95 - 1.01) | (0.95 - 1.01) |
| Area lived in (ref: big city) |  |  |
| Suburbs or outskirts of a big city | 1.92 | 1.88 |
|  | (0.85 - 4.33) | (0.85 - 4.15) |
| Large town | 1.43 | 1.42 |
|  | (0.61 - 3.33) | (0.63 - 3.24) |
| Small town | 1.24 | 1.21 |
|  | (0.63 - 2.45) | (0.62 - 2.35) |
| Rural area or village | 1.35 | 1.31 |
|  | (0.70 - 2.59) | (0.69 - 2.48) |
| Living with partner in the same household (ref: no) | 1.00 | 0.99 |
|  | (0.62 - 1.63) | (0.62 - 1.60) |
| Employment situation (ref: retired) |  |  |
| Employed or self-employed | 0.58 | 0.59 |
|  | (0.29 - 1.13) | (0.30 - 1.16) |
| Other | 0.80 | 0.83 |
|  | (0.35 - 1.84) | (0.37 - 1.87) |
| Households’ ability to make ends meet (ref: easily) |  |  |
| With great or some difficulty | 0.71 | 0.75 |
|  | (0.31 - 1.63) | (0.34 - 1.67) |
| Fairly easily | 0.72 | 0.73 |
|  | (0.45 - 1.15) | (0.46 - 1.16) |
| Sad or depressed in the last month (ref: no) | 1.64* | 1.62* |
|  | (1.05 - 2.56) | (1.05 - 2.51) |
| Frequency of social contacts with nonrelatives (ref: never) |  |  |
| Less than once a week | 1.19 | 1.16 |
|  | (0.60 - 2.40) | (0.59 - 2.29) |
| About once a week | 0.86 | 0.85 |
|  | (0.41 - 1.84) | (0.41 - 1.78) |
| Several times a week | 1.14 | 1.12 |
|  | (0.54 - 2.41) | (0.54 - 2.32) |
| Daily | 0.88 | 0.87 |
|  | (0.37 - 2.09) | (0.38 - 2.04) |
| Frequency of electronic contacts with nonrelatives (ref: never) |  |  |
| Less than once a week | 1.04 | 1.02 |
|  | (0.50 - 2.17) | (0.50 - 2.08) |
| About once a week | 1.50 | 1.44 |
|  | (0.72 - 3.12) | (0.71 - 2.95) |
| Several times a week | 1.23 | 1.20 |
|  | (0.58 - 2.61) | (0.57 - 2.49) |
| Daily | 1.23 | 1.24 |
|  | (0.46 - 3.31) | (0.48 - 3.22) |
| Having trouble sleeping recently (ref: no) | 0.83 | 0.84 |
|  | (0.54 - 1.29) | (0.55 - 1.29) |
| Number of physical illnesses (range 0-6) | 1.02 | 1.02 |
|  | (0.83 - 1.26) | (0.83 - 1.26) |
| Health limitations (ref: not limited) |  |  |
| Severely limited | 2.17* | 2.14* |
|  | (1.14 - 4.14) | (1.13 - 4.04) |
| Limited, but not severely | 1.00 | 1.01 |
|  | (0.61 - 1.66) | (0.61 - 1.66) |
| Self-rated health (ref: excellent) |  |  |
| Very good | 1.63 | 1.36 |
|  | (0.36 - 7.38) | (0.35 - 5.33) |
| Good | 1.88 | 1.54 |
|  | (0.44 - 8.09) | (0.41 - 5.71) |
| Fair | 1.92 | 1.57 |
|  | (0.42 - 8.70) | (0.40 - 6.17) |
| Poor | 1.23 | 1.03 |
|  | (0.23 - 6.61) | (0.22 - 4.85) |
| Received COVID-19 vaccine (ref: no) | 0.77 | 0.76 |
|  | (0.40 - 1.49) | (0.40 - 1.43) |
| Self or anyone close tested positive for COVID-19 (ref: no) | 1.00 | 1.01 |
|  | (0.65 - 1.54) | (0.66 - 1.54) |
| Forgone medical treatment since COVID-19 pandemic (ref: no) | 1.86* | 1.86* |
|  | (1.12 - 3.10) | (1.13 - 3.05) |
| Took drugs/medicine as prevention against COVID-19 (ref: no) | 1.06 | 1.21 |
|  | (0.31 - 3.63) | (0.39 - 3.76) |
| Constant | 0.07+ | 0.10 |
|  | (0.00 - 1.48) | (0.01 - 1.87) |
|  |  |  |
| Observations | 1,973 | 1,973 |
| Pseudo R² | 0.045 | 0.052 |
| Likelihood ratio chi-square test statistic | 39.12 |  |
| Wald chi-square test statistic |  |  |

*Note.* Odds Ratios are reported with 95% confidence intervals in parentheses. * p<0.05, + p<0.10.

**Table S3. Results from logistic regression and Firth logistic regression for determinants of telemedicine service use during the COVID-19 pandemic (with feelings of loneliness).**

|  | Results from | Results from |
| --- | --- | --- |
| Independent Variables | Logistic Regression | Firth Logistic Regression |
|  |  |  |
| Female sex (ref: male) | 1.35 | 1.34 |
|  | (0.89 - 2.05) | (0.89 - 2.02) |
| Age | 0.98 | 0.98 |
|  | (0.95 - 1.01) | (0.95 - 1.01) |
| Area lived in (ref: big city) |  |  |
| Suburbs or outskirts of a big city | 1.80 | 1.77 |
|  | (0.80 - 4.07) | (0.80 - 3.91) |
| Large town | 1.39 | 1.39 |
|  | (0.60 - 3.23) | (0.61 - 3.15) |
| Small town | 1.20 | 1.17 |
|  | (0.61 - 2.37) | (0.60 - 2.28) |
| Rural area or village | 1.32 | 1.29 |
|  | (0.69 - 2.54) | (0.68 - 2.44) |
| Living with partner in the same household (ref: no) | 1.07 | 1.06 |
|  | (0.65 - 1.75) | (0.65 - 1.71) |
| Employment situation (ref: retired) |  |  |
| Employed or self-employed | 0.59 | 0.61 |
|  | (0.30 - 1.17) | (0.31 - 1.19) |
| Other | 0.83 | 0.87 |
|  | (0.36 - 1.92) | (0.39 - 1.95) |
| Households’ ability to make ends meet (ref: easily) |  |  |
| With great or some difficulty | 0.69 | 0.72 |
|  | (0.30 - 1.57) | (0.32 - 1.61) |
| Fairly easily | 0.73 | 0.74 |
|  | (0.46 - 1.17) | (0.47 - 1.18) |
| Feelings of loneliness in the last month (ref: no) | 1.68* | 1.66* |
|  | (1.07 - 2.62) | (1.07 - 2.58) |
| Frequency of social contacts with nonrelatives (ref: never) |  |  |
| Less than once a week | 1.19 | 1.16 |
|  | (0.60 - 2.38) | (0.59 - 2.27) |
| About once a week | 0.85 | 0.84 |
|  | (0.40 - 1.81) | (0.40 - 1.75) |
| Several times a week | 1.15 | 1.13 |
|  | (0.55 - 2.43) | (0.55 - 2.32) |
| Daily | 0.89 | 0.88 |
|  | (0.37 - 2.11) | (0.38 - 2.06) |
| Frequency of electronic contacts with nonrelatives (ref: never) |  |  |
| Less than once a week | 1.04 | 1.02 |
|  | (0.50 - 2.17) | (0.50 - 2.07) |
| About once a week | 1.54 | 1.48 |
|  | (0.74 - 3.20) | (0.73 - 3.02) |
| Several times a week | 1.25 | 1.22 |
|  | (0.59 - 2.66) | (0.58 - 2.53) |
| Daily | 1.24 | 1.24 |
|  | (0.46 - 3.31) | (0.48 - 3.21) |
| Having trouble sleeping recently (ref: no) | 0.89 | 0.90 |
|  | (0.58 - 1.36) | (0.59 - 1.36) |
| Number of physical illnesses (range 0-6) | 1.02 | 1.02 |
|  | (0.82 - 1.26) | (0.83 - 1.25) |
| Health limitations (ref: not limited) |  |  |
| Severely limited | 2.23* | 2.20* |
|  | (1.17 - 4.25) | (1.17 - 4.15) |
| Limited, but not severely | 1.00 | 1.01 |
|  | (0.61 - 1.66) | (0.61 - 1.66) |
| Self-rated health (ref: excellent) |  |  |
| Very good | 1.60 | 1.34 |
|  | (0.35 - 7.25) | (0.34 - 5.24) |
| Good | 1.80 | 1.47 |
|  | (0.42 - 7.75) | (0.40 - 5.48) |
| Fair | 1.94 | 1.58 |
|  | (0.43 - 8.78) | (0.40 - 6.23) |
| Poor | 1.34 | 1.13 |
|  | (0.25 - 7.17) | (0.24 - 5.26) |
| Received COVID-19 vaccine (ref: no) | 0.81 | 0.80 |
|  | (0.42 - 1.58) | (0.42 - 1.52) |
| Self or anyone close tested positive for COVID-19 (ref: no) | 1.01 | 1.01 |
|  | (0.66 - 1.54) | (0.67 - 1.55) |
| Forgone medical treatment since COVID-19 pandemic (ref: no) | 1.86* | 1.85* |
|  | (1.12 - 3.09) | (1.13 - 3.04) |
| Took drugs/medicine as prevention against COVID-19 (ref: no) | 1.05 | 1.19 |
|  | (0.31 - 3.58) | (0.38 - 3.70) |
| Constant | 0.06+ | 0.08+ |
|  | (0.00 - 1.24) | (0.00 - 1.58) |
|  |  |  |
| Observations | 1,973 | 1,973 |
| Pseudo R² | 0.045 | 0.052 |
| Likelihood ratio chi-square test statistic | 39.18 |  |
| Wald chi-square test statistic |  | 39.79 |

*Note.* Odds Ratios are reported with 95% confidence intervals in parentheses. * p<0.05, + p<0.10.

**Table S4. Results from Firth logistic regression for all models including age as categorical variable.**

|  | | | Model with | | | | Model with | | | Model with | | | | | | | | |
| --- | --- | --- | --- | --- | --- | --- | --- | --- | --- | --- | --- | --- | --- | --- | --- | --- | --- | --- |
| Independent Variables | | | Anxiety  Symptoms | | | | Depressive Symptoms | | | Feelings of Loneliness | | | | | | | | |
|  | | |  | | | |  | | |  | | | | | | | | |
| Female sex (ref: male) | | | 1.31 | | | | 1.32 | | | 1.34 | | | | | | | | |
|  | | | (0.87 - 1.98) | | | | (0.87 - 1.99) | | | (0.89 - 2.02) | | | | | | | | |
| Age (ref: 40-64 years) | | |  | | | |  | | |  | | | | | | | | |
| 65-74 years | | | 0.43* | | | | 0.43* | | | 0.46* | | | | | | | | |
|  | | | (0.22 - 0.86) | | | | (0.22 - 0.86) | | | (0.23 - 0.91) | | | | | | | | |
| ≥75 years | | | 0.38* | | | | 0.37* | | | 0.40* | | | | | | | | |
|  | | | (0.17 - 0.86) | | | | (0.17 - 0.84) | | | (0.18 - 0.90) | | | | | | | | |
| Area lived in (ref: big city) | | |  | | | |  | | |  | | | | | | | | |
| Suburbs or outskirts of a big city | | | 1.92 | | | | 1.91 | | | 1.79 | | | | | | | | |
|  | | | (0.87 - 4.23) | | | | (0.86 - 4.21) | | | (0.81 - 3.96) | | | | | | | | |
| Large town | | | | | | 1.47 | | | | 1.49 | | | | | 1.43 | | | |
|  | | | | | | (0.65 - 3.35) | | | (0.65 - 3.38) | | | | (0.63 - 3.25) | | | | | |
| Small town | | | | | | 1.19 | | | 1.20 | | | | 1.16 | | | | | |
|  | | | | | | (0.61 - 2.33) | | | (0.62 - 2.34) | | | | (0.60 - 2.26) | | | | | |
| Rural area or village | | | | | | 1.30 | | | | | 1.30 | | | | 1.27 | | | |
|  | | | | (0.69 - 2.45) | | | | | | | (0.69 - 2.46) | | | | | | (0.67 - 2.40) | |
| Living with partner in the same household | | | 0.93 | | | | 0.98 | | | 1.04 | | | | | | | | |
|  | | | (0.58 - 1.50) | | | | (0.61 - 1.57) | | | (0.64 - 1.68) | | | | | | | | |
| Employment situation (ref: retired) | | |  | | | |  | | |  | | | | | | | | |
| Employed or self-employed | | | 0.36* | | | | 0.36* | | | 0.39* | | | | | | | | |
|  | | | (0.16 - 0.79) | | | | (0.17 - 0.79) | | | (0.18 - 0.85) | | | | | | | | |
| Other | | | 0.52 | | | | 0.53 | | | 0.58 | | | | | | | | |
|  | | | (0.21 - 1.29) | | | | (0.21 - 1.29) | | | (0.24 - 1.40) | | | | | | | | |
| Households’ ability to make ends meet (ref: easily) | | |  | | | |  | | |  | | | | | | | | |
| With great or some difficulty | | | 0.73 | | | | 0.75 | | | 0.72 | | | | | | | | |
|  | | | (0.33 - 1.63) | | | | (0.34 - 1.66) | | | (0.32 - 1.61) | | | | | | | | |
| Fairly easily | | | 0.73 | | | | 0.73 | | | 0.74 | | | | | | | | |
|  | | | (0.46 - 1.16) | | | | (0.46 - 1.15) | | | (0.47 - 1.17) | | | | | | | | |
| Nervous, anxious, or on edge in the last month (ref: no) | | | 1.64* | | | |  | | |  | | | | | | | | |
|  | | | (1.05 - 2.56) | | | |  | | |  | | | | | | | | |
| Sad or depressed in the last month (ref: no) | | |  | | | | 1.65* | | |  | | | | | | | | |
|  | | |  | | | | (1.06 - 2.56) | | |  | | | | | | | | |
| Feelings of loneliness in the last month (ref: no) | | |  | | | |  | | | 1.64* | | | | | | | | |
|  | | |  | | | |  | | | (1.06 - 2.55) | | | | | | | | |
| Frequency of social contacts with nonrelatives (ref: never) | | |  | | | |  | | |  | | | | | | | | |
| Less than once a week | | | 1.12 | | | | 1.13 | | | 1.14 | | | | | | | | |
|  | | | (0.56 - 2.21) | | | | (0.57 - 2.24) | | | (0.58 - 2.24) | | | | | | | | |
| About once a week | | | 0.87 | | | | 0.86 | | | 0.85 | | | | | | | | |
|  | | | (0.41 - 1.82) | | | | (0.41 - 1.80) | | | (0.41 - 1.78) | | | | | | | | |
| Several times a week | | | 1.10 | | | | 1.09 | | | 1.10 | | | | | | | | |
|  | | | (0.53 - 2.28) | | | | (0.53 - 2.26) | | | (0.53 - 2.28) | | | | | | | | |
| Daily | | | 0.85 | | | | 0.84 | | | 0.85 | | | | | | | | |
|  | | | (0.36 - 1.98) | | | | (0.36 - 1.96) | | | (0.36 - 1.98) | | | | | | | | |
| Frequency of electronic contacts with nonrelatives (ref: never) | | |  | | | |  | | |  | | | | | | | | |
| Less than once a week | | | 1.07 | | | | 1.04 | | | 1.04 | | | | | | | | |
|  | | | (0.53 - 2.19) | | | | (0.51 - 2.12) | | | (0.51 - 2.11) | | | | | | | | |
| About once a week | | | 1.47 | | | | 1.44 | | | 1.46 | | | | | | | | |
|  | | | (0.72 - 3.01) | | | | (0.70 - 2.94) | | | (0.72 - 2.99) | | | | | | | | |
| Several times a week | | | 1.26 | | | | 1.24 | | | 1.26 | | | | | | | | |
|  | | | (0.60 - 2.63) | | | | (0.60 - 2.60) | | | (0.60 - 2.62) | | | | | | | | |
| Daily | | | 1.25 | | | | 1.25 | | | 1.25 | | | | | | | | |
|  | | | (0.49 - 3.25) | | | | (0.48 - 3.25) | | | (0.48 - 3.22) | | | | | | | | |
| Having trouble sleeping recently (ref: no) | | | 0.84 | | | | 0.83 | | | 0.89 | | | | | | | | |
|  | | | (0.54 - 1.28) | | | | (0.54 - 1.28) | | | (0.58 - 1.35) | | | | | | | | |
| Number of physical illnesses (range 0-6) | | | 1.03 | | | | 1.02 | | | 1.02 | | | | | | | | |
|  | | | (0.83 - 1.27) | | | | (0.83 - 1.26) | | | (0.83 - 1.25) | | | | | | | | |
| Health limitations (ref: not limited) | | |  | | | |  | | |  | | | | | | | | |
| Severely limited | | | 2.12* | | | | 2.14* | | | 2.21* | | | | | | | | |
|  | | | (1.12 - 4.02) | | | | (1.13 - 4.05) | | | (1.17 - 4.17) | | | | | | | | |
| Limited, but not severely | | | 1.01 | | | | 1.02 | | | 1.03 | | | | | | | | |
|  | | | (0.61 - 1.66) | | | | (0.62 - 1.68) | | | (0.62 - 1.69) | | | | | | | | |
| Self-rated health (ref: excellent) | | |  | | | |  | | |  | | | | | | | | |
| Very good | | | 1.41 | | | | 1.39 | | | 1.37 | | | | | | | | |
|  | | | (0.36 - 5.53) | | | | (0.35 - 5.48) | | | (0.35 - 5.39) | | | | | | | | |
| Good | | | 1.56 | | | | 1.58 | | | 1.51 | | | | | | | | |
|  | | | (0.42 - 5.78) | | | | (0.42 - 5.88) | | | (0.41 - 5.63) | | | | | | | | |
| Fair | | | 1.57 | | | | 1.58 | | | 1.60 | | | | | | | | |
|  | | | (0.40 - 6.19) | | | | (0.40 - 6.26) | | | (0.41 - 6.30) | | | | | | | | |
| Poor | | | 1.05 | | | | 1.00 | | | 1.11 | | | | | | | | |
|  | | | (0.22 - 4.93) | | | | (0.21 - 4.74) | | | (0.24 - 5.18) | | | | | | | | |
| Received COVID-19 vaccine (ref: no) | | | 0.74 | | | | 0.76 | | | 0.80 | | | | | | | | |
|  | | | (0.39 - 1.39) | | | | (0.40 - 1.44) | | | (0.42 - 1.52) | | | | | | | | |
| Self or anyone close tested positive for COVID-19 (ref: no) | | | 0.99 | | | | 0.99 | | | 1.00 | | | | | | | | |
|  | | | (0.65 - 1.51) | | | | (0.65 - 1.51) | | | (0.66 - 1.52) | | | | | | | | |
| Forgone medical treatment since COVID-19 pandemic (ref: no) | | | 1.74* | | | | 1.79* | | | 1.79* | | | | | | | | |
|  | | | (1.06 - 2.86) | | | | (1.09 - 2.94) | | | (1.09 - 2.94) | | | | | | | | |
| Took drugs/medicine as prevention against COVID-19 (ref: no) | | | 1.25 | | | | 1.19 | | | 1.20 | | | | | | | | |
|  | | | (0.40 - 3.88) | | | | (0.38 - 3.75) | | | (0.39 - 3.74) | | | | | | | | |
| Constant | | | 0.06** | | | | 0.06** | | | 0.05** | | | | | | | | |
|  | | | (0.01 - 0.40) | | | | (0.01 - 0.37) | | | (0.01 - 0.33) | | | | | | | | |
|  | | |  | | | |  | | |  | | | | | | | | |
| Observations | | | 1,976 | | | | 1,973 | | | 1,973 | | | | | | | | |
| Pseudo R² | | | 0.058 | | | | 0.058 | | | 0.058 | | | | | | | | |
| Wald chi-square test statistic | | | 43.81+ | | | | 44.19+ | | | 44.19+ | | | | | | | | |

*Note.* Odds Ratios are reported with 95% confidence intervals in parentheses. ** p<0.01, * p<0.05, + p<0.10.
